# Granulomatous rosacea treated with tapinarof

**DOI:** 10.1016/j.jdcr.2025.06.038

**Published:** 2025-07-11

**Authors:** Mitchell Brady, Vera Wang, Francesca Kartono, Lynn Sikorski

**Affiliations:** a Dermatology, Corewell Health Farmington Hills, Farmington Hills, Michigan; b Western University of Health Sciences, Pomona, California; c Dermatology, Corewell Health Farmington Hills, Farmington Hills, Michigan

**Keywords:** granulomatous, novel, rosacea, tapinarof, Vtama

## Introduction

Granulomatous rosacea (GR) is a rare variant of rosacea marked by granulomatous inflammation. Clinically, it manifests as persistent erythema, papules, and yellowish brown hard nodules, most commonly affecting the forehead. It can also affect the periocular and perioral areas, and is typically seen in middle-aged women. Histologically, GR is defined by granulomatous infiltrates consisting primarily of lymphocytes, histiocytes, and multinucleated giant cells.^1^

The exact pathogenesis of GR remains unclear, though it is believed to involve immune dysregulation and antigenic triggers leading to granuloma formation.^2^ Treatment of GR is difficult and often involves a combination of topical and systemic therapies.^2^ Tetracyclines are often used for their anti-inflammatory properties; however, there is no standard treatment.^3^, ^4^, ^5^

Tapinarof is a topical, nonsteroidal, aryl hydrocarbon receptor agonist typically used to treat plaque psoriasis.^6^ It has been approved by the US Food and Drug Administration for the treatment of plaque psoriasis in adults and is under investigation for atopic dermatitis. We report a case of a patient with GR refractory to initial treatments that later resolved with tapinarof.

## Case report

**Fig 1:**
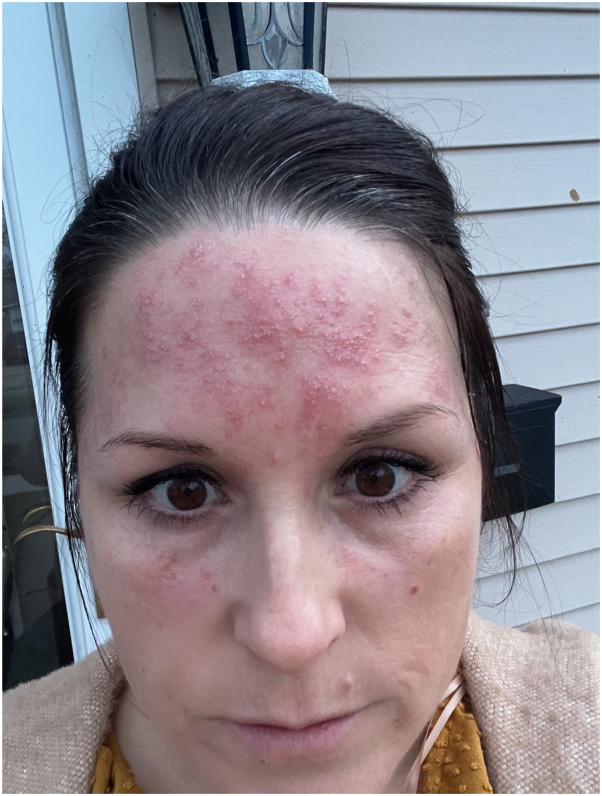
Initial presentation of granulomatous rosacea (GR) with erythematous papules and pustules on the forehead and cheeks.

A 44-year-old female presented to our clinic 6-8 months after developing a facial rash (Fig 1). Her past medical history included only eczema. On examination, erythematous papules and pustules coalescing into plaques on her forehead and cheeks were noted. The patient’s prior dermatologist prescribed triamcinolone and oral ivermectin, without improvement. At our clinic, the patient was started on betamethasone twice a day, tacrolimus once a day, and doxycycline, and labs were ordered. The differential diagnosis at this time was cutaneous lupus versus rosacea. At follow-up, the patients’ labs, including free thyroxine, thyroid-stimulating hormone, complete blood count, antinuclear antibody, antinuclear antibody titer and pattern, anti–Sjögren syndrome-related antigen A antibody, anti–Sjögren syndrome-related antigen B antibody, anti-double-stranded DNA antibody, and anti-Smith antibody, were all unremarkable.

Punch biopsies of the upper and lower forehead were performed 2 months after her initial presentation for definitive diagnosis of lupus versus rosacea. Upper forehead biopsy demonstrated granulomatous inflammation evidenced by histiocytes, some multinucleate, and lymphocytes. The inflammation shows a folliculocentric distribution with neutrophils and eosinophils, consistent with GR. No interface dermatitis, classically seen in lupus, was present, and infectious microorganisms were not seen with special stains. There was also no evidence of connective tissue disease, vasculitis, or immunobullous disorder.

**Fig 2:**
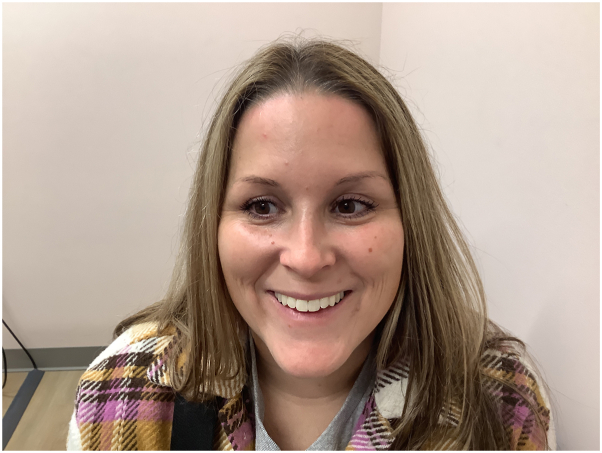
Granulomatous rosacea (GR) cleared after 4 months of tapinarof treatment.

The patient was started on tapinarof twice a day, 1 month after her biopsy was performed, and her GR fully cleared 4 months later (Fig 2). The patient has continued using tapinarof and her symptoms have not returned.

## Discussion

There is currently no standard treatment for GR due to the rare and refractory nature of the disease. The pathophysiology of GR remains unclear; however, it is likely to involve immune dysregulation, with key contributing factors including increased neutrophil activity and collagen hyperplasia. GR demonstrates higher infiltration of CD4, CD8, and CD68 cells compared to non-GR, possibly playing a role in macrophage recruitment to form granulomas.^7^ Furthermore, matrix metalloproteinases (MMPs) associated with ultraviolet radiation, especially MMP-9, are significantly upregulated in GR lesions, implicating them in granuloma formation and tissue remodeling.^8^

Although tapinarof is primarily used for psoriasis, its anti-inflammatory actions may make it effective in treating GR. Tapinarof binds to aryl hydrocarbon receptor, a ligand-dependent transcription factor, leading to the downregulation of proinflammatory cytokines and mast cell degranulation markers.^9^ One study demonstrates that tapinarof specifically reduces LL37, which results in reduction of Chymase 1, Tryptase-alpha/beta1, MMP9, tumor necrosis factor-α, and interleukin-6 in mast cells. These markers promote the pathogenesis of rosacea, so tapinarof’s role in downregulating them may explain its success in treating this patient with GR refractory to triamcinolone, ivermectin, betamethasone, tacrolimus, and doxycycline.^10^ There are currently no other reported cases of GR resolved with tapinarof. Larger controlled studies are necessary to further evaluate tapinarof’s efficacy and safety for GR treatment.

## Conflicts of interest

None disclosed.
